# 离子色谱-脉冲安培法测定空气中微量硫化氢

**DOI:** 10.3724/SP.J.1123.2023.10028

**Published:** 2024-08-08

**Authors:** Xiaojing GAO, Tong NI, Rui SHEN, Chaoou SHI

**Affiliations:** 华东理工大学化学与分子工程学院分析测试中心, 上海 200237; Analysis and Testing Center, School of Chemistry and Molecular Engineering, East China University of Science and Technology, Shanghai 200237, China

**Keywords:** 离子色谱, 安培检测, 硫化物, 电位, 草酸钠, ion chromatography (IC), amperometric detection, sulfide, potential, sodium oxalate

## Abstract

硫化氢(H_2_S)是无色有毒气体,低浓度即有臭鸡蛋气味,浓度足够高时会麻痹嗅觉神经反而不易被察觉,人体长时间接触H_2_S会使呼吸系统和中枢神经等受到严重损害。低浓度硫化氢可用离子色谱法检测,针对现有方法存在的问题,本文建立了一种准确、高效测定微量硫化氢气体的被动采样-离子色谱-脉冲安培定量分析方法。该法使用经典的IonPac AS7 (250 mm×4 mm)阴离子交换色谱柱,并采用新型氢氧化钠-草酸钠淋洗液组合替代传统方法中氢氧化钠-乙酸钠淋洗液组合,优化脉冲安培检测电位参数,实现了低浓度硫化物的检测;探究提取稳定液的种类和含量对硫化物稳定性的影响,建立了硫化物的最佳保存条件。实验结果表明,硫化物在10~3000 μg/L的范围内线性关系良好(相关系数*r*^2^>0.999),检出限(*S/N*=3)和定量限(*S/N*=10)分别为1.53 μg/L和5.10 μg/L,保留时间和峰面积的相对标准偏差(RSD)均小于0.2% (*n*=6)。改进后方法稳定性好,操作要求低,硫化物峰形更为对称,基线噪声显著降低,且淋洗液试剂成本只有原来的10%,更适用于实际样品中低浓度硫化物的检测。采用250 mmol/L氢氧化钠-0.8%(质量分数)乙二胺四乙酸二钠稳定液可使硫化物稳定保存10 h以上,提高了回收率,使大批量、长时间检测更具可靠性。将新建立的方法应用于学校垃圾处理站附近空气中硫化氢含量的测定,检测结果均未超过国家规定限值,此法可满足硫化氢气体相关鉴定检测工作的要求。

## Abstract (en)

Hydrogen sulfide (H_2_S) is a pervasive gaseous pollutant that emits the characteristic odor of rotten gas, even at low concentrations. It is generated during various industrial processes, including petroleum and natural gas refining, mining operations, wastewater treatment activities, and refuse disposal practices. According to statistics from the World Health Organization (WHO), over 70 occupations are exposed to H_2_S, rendering it a key monitoring factor in occupational disease detection. Although H_2_S has legitimate uses in the chemical, medical, and other fields, prolonged exposure to this gas can cause severe damage to the respiratory and central nervous systems, as well as other organs in the human body. Moreover, the substantial release of H_2_S into the environment can lead to significant pollution. This noxious substance has the potential to impair soil, water, and air quality, while disrupting the equilibrium of the surrounding ecosystems. Therefore, sulfide has become one of the most commonly measured substances for environmental monitoring worldwide. Achieving the stable enrichment and accurate detection of low-level H_2_S is of great significance. Common methods for detecting this gas include spectrophotometry, chemical analysis, gas chromatography, rapid field detection, and ion chromatography. Although these methods provide relatively reliable results, they suffer from limitations such as high detection cost, low recovery, lack of environmental friendliness, and imprecise quantification of low-concentration H_2_S. Furthermore, the sampling processes involved in these methods are complex and require specialized equipment and electrical devices. Additionally, approximately 20% of the sulfides in a sample are lost after 2 h in a conventional alkaline sodium hydroxide solution, causing difficulties in preservation and detection.

In this study, an accurate, efficient, and cost-saving method based on ion chromatography-pulse amperometry was developed for H_2_S determination. A conventional IonPac AS7 (250 mm×4 mm) anion-exchange column was employed, and a new eluent based on sodium hydroxide and sodium oxalate was used to replace the original sodium hydroxide-sodium acetate eluent. The main factors influencing the separation and detection performance of the proposed method, including the pulse amperage detection potential parameters and integration time, as well as the type and content of additives in the stabilizing solution, were optimized. The results showed that the proposed method had a good linear relationship between 10 and 3000 μg/L, with correlation coefficients (*r*^2^) of up to 0.999. The limits of detection (*S/N*=3) and quantification (*S/N*=10) were 1.53 and 5.10 μg/L, respectively. The relative standard deviations (RSDs) of the peak area and retention time of sulfides were less than 0.2% (*n*=6). The new method exhibited excellent stability, with up to 90% reduction in reagent costs. Compared with conventional ion chromatography-pulse amperometry, this method is more suitable for detecting low concentrations of sulfides in actual samples. Sulfides in a 250 mmol/L sodium hydroxide-0.8% (mass fraction) ethylenediaminetetraacetic acid disodium salt solution were effectively maintained for over 10 h. The new stabilizer significantly improved the reliability of both large-scale and long-term detection. The recovery of the proposed method was investigated by combining the system with a badge-type passive sampler. This sampling method requires no power devices; it is inexpensive, simple to operate, and can realize long-term sampling without the need for skilled personnel. Moreover, it can overcome the influence of short-term changes in pollutant concentration. The sampling results have high reference value for large-scale intervention-less pollutant monitoring in ultraclean rooms, museum counters, and other places. The results demonstrated that the recovery of the proposed method was greater than 95% for the blank sample and 80% for the sample plus standard solution. Finally, the newly established method was applied to determine H_2_S levels in air samples collected via passive sampling at school garbage stations. The measured results did not exceed the national limit.

硫化氢(H_2_S)是人类生产生活中常见的气态污染物,低浓度即具有臭鸡蛋气味,浓度足够高时能麻痹嗅觉神经反而不易被察觉。采矿、造纸、石油和天然气加工、废水处理、垃圾处理等过程均会产生硫化氢气体,其来源广泛,控制困难。人体长期暴露于硫化氢环境中会产生头晕、恶心等症状,大量吸入还会造成呼吸衰竭而迅速死亡^[1,2]^;硫化氢也是形成酸雨的原因之一,被氧化后形成的硫酸会腐蚀植物根茎和室外管道,对自然环境和人类生产生活产生恶劣影响^[3,4]^。

近年来,检测硫化氢的常用方法有分光光度法^[5]^、化学分析法^[6]^、气相色谱法^[7,8]^、离子色谱法^[9,10]^、现场快速检测法(如醋酸铅试纸、硫化氢报警法)^[11]^等。丁萌萌等^[12]^建立了电子制冷预浓缩仪-气相色谱-质谱法测定空气中10种含硫化合物的方法,得到硫化氢方法检出限为1.1 μg/m^3^。李媛等^[13]^采用冷冻聚焦前处理技术和气相色谱-硫发光检测器测定环境空气与无组织排放空气中硫化氢等16种含硫化合物,得到硫化氢的方法检出限为0.14 μg/m^3^。张妍等^[14]^用装有氢氧化钠溶液的多孔玻板吸收管采集工作场所空气中的硫化氢,采用离子色谱检测,计算得到方法检出限,为4.7 μg/m^3^。上述方法虽能提供较可靠的检测结果,但硫化氢富集采样困难、样品前处理复杂、回收率低且检测成本高。近年来,离子色谱法高效、简便、灵敏等优点使其逐渐应用到硫化氢的测定工作中。使用离子色谱法将富集的硫化氢以离子形式分离,直流或脉冲安培法检测电流变化,反映出硫化氢的浓度^[15]^。但在日常检测中发现,硫化物在常规氢氧化钠碱性溶液中保存2 h就会损失20%左右,保存困难,易被氧化;且现有方法对实验条件要求高,方法重现性不佳,低浓度硫化物峰严重拖尾或不能检出,被动采样的回收率仅有60%左右,给实际应用带来重重困难。

本文使用徽章式被动采样器^[16]^富集空气中的硫化氢,无需动力装置,成本较低,操作简单,可克服短期内污染物浓度变化的影响,对于超净室、博物馆柜台、展厅等场所进行大规模无介入式污染物监测更具参考价值。本方法在原有银电极测HS^-[17]^的基础上优化淋洗液条件,降低了基线噪声,分离效果更好,大大降低了成本;优化脉冲安培检测电位,在默认硫化物电位的基础上,针对低浓度硫化物优化电位参数,探索积分时长的影响。本研究建立了一套准确、可靠地测定低浓度硫化物的离子色谱方法,检出限、定量限更低,对比加入几种稳定液后硫化物稳定存在时间,得到硫化物的最佳保存条件,提高了硫化物的回收率。本方法成功应用于学校垃圾处理站的实际样品检测。

## 1 实验部分

### 1.1 仪器与试剂

ICS5000+离子色谱仪,配备安培检测器和自动进样器(美国Thermo Fisher公司); KQ5200DA型数控超声波清洗器(昆山市超声仪器有限公司); Milli-QA10超纯水仪(美国Millipore公司); 50%(质量分数)氢氧化钠溶液(NaOH,分析纯)、乙二胺(EDA,纯度99%)均购自赛默飞世尔科技中国有限公司;草酸钠(Na_2_C_2_O_4_,纯度99.5%)、乙酸钠(NaAc,纯度99%)均购自德国Sigma-Aldrich公司;乙二胺四乙酸二钠(EDTA,纯度99.99%)、硫化物标准溶液(S^2-^,100 mg/L)均购自上海麦克林生化科技公司。

### 1.2 溶液的配制

250 mmol/L NaOH-0.8%(质量分数)EDTA稳定液:称取2.0 g 50%氢氧化钠溶液、0.8 g乙二胺四乙酸二钠于100 mL容量瓶中,加入煮沸后冷却的超纯水,定容摇匀。

200 mmol/L NaOH-7.5 mmol/L Na_2_C_2_O_4_淋洗液:称取2.0 g草酸钠、32.0 g 50%氢氧化钠溶液于流动相瓶中,加入超纯水至2 L,超声30 min。

标准溶液:准确移取100 mg/L硫化物标准溶液,使用250 mmol/L NaOH-0.8% EDTA稳定液稀释,配制成所需浓度的系列标准溶液,于4 ℃冷藏保存,现用现配。S^2-^在9<pH<13的碱性溶液中以HS^-^状态存在,下文均用硫化物代称。

### 1.3 色谱条件

色谱柱:IonPac AS7 (250 mm×4 mm,美国Thermo Fisher公司);保护柱:IonPac AG7 (50 mm×4 mm,美国Thermo Fisher公司);检测模式:安培检测;工作电极:银电极;参比电极:Ag/AgCl-pH复合参比电极;电位波形见表1;淋洗液:200 mmol/L NaOH-7.5 mmol/L Na_2_C_2_O_4_;流速:1 mL/min;柱温:30 ℃;进样量:25 μL。

**表1:** 电位波形参数

Time/s	Potential (vs Ag/AgCl)/mV	Integration
0	-0.1	
0.20	-0.1	begin
0.90	-0.1	end
0.91	0.1	
1.00	0.1	

### 1.4 样品采集及预处理

硫化氢的采集使用徽章式被动采样器^[16]^,由外壳、挡风网、防尘防风滤膜、连接件、滤纸、吸收液组成。采样时于滤纸上滴加100 μL吸收液后组装,放置于采样地点即开始采样,72 h后取出底盖中的滤纸和滤膜,加入5 mL 250 mmol/L NaOH-0.8% EDTA稳定液,超声提取15 min,溶液过0.45 μm过滤器后直接进样分析。

### 1.5 空气中硫化氢含量的计算

被动采样器依靠硫化氢分子扩散至吸收液发生反应完成污染气体的采集。气体吸收系数与采样器结构和环境因素有关,在环境稳定的情况下,同种采样器的吸收系数为定值。此时,采样结果仅与空气中硫化氢的含量和采样的时间呈正比,空气中硫化氢含量的计算公式如下:




*C=m*_s_/*at*

式中,*t*为被动采样的时间,h; *C*为硫化氢的平均含量,μg/m^3^; *m*_s_为采集到硫化氢气体的总质量,μg; *a*为被动采样的吸收系数,为1.1836×10^-4^m^3^/h。

## 2 结果与讨论

### 2.1 色谱条件的优化

#### 2.1.1 流动相类型的选择

目前硫化物检测常用的淋洗液由氢氧化钠和乙酸钠组成。由于电化学方法的特殊性,离子色谱使用的乙酸钠对金属离子等杂质含量控制较严,目前国内无法生产,全部依赖进口。此外,淋洗液中乙酸钠浓度较大,会导致基线背景相对较高且使用成本偏高。为解决以上问题,尝试使用低浓度草酸钠替代高浓度乙酸钠。草酸钠为二价离子,洗脱强度大于乙酸钠,且能掩蔽重金属离子,可降低基线噪声,并减少金属离子对硫化物测定的影响。实验考察了淋洗液中加入乙二胺掩蔽重金属离子后对硫化物测定的影响。使用原有硫化物测定方法(电位波形见表2,其他条件见1.3节),考察3种淋洗液(200 mmol/L NaOH-7.5 mmol/L Na_2_C_2_O_4_、100 mmol/L NaOH-500 mmol/L NaAc、100 mmol/L NaOH-500 mmol/L NaAc-0.5%(质量分数) EDA)对硫化物测定的影响(见图1)。

**表2:** IonPac AS7色谱柱分析硫化物的默认电位波形参数

Time/s	Potential (vs Ag/AgCl)/mV	Integration
0	-0.1	
0.20	-0.1	begin
0.90	-0.1	end
0.91	-1.0	
0.93	-0.3	
1.00	-0.3	

**图1:**
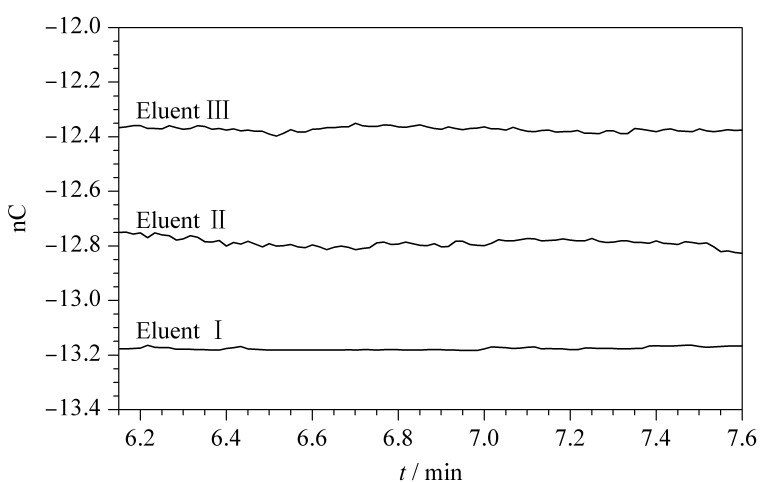
3种淋洗液对基线噪声的影响

实验对500 μg/L硫化物进行检测,发现淋洗液中加入乙二胺后,硫化物峰形没有改善。低浓度草酸钠代替高浓度的乙酸钠,基线噪声从0.06 nC降低到0.01 nC(见图1),且有非常好的柱效和选择性。草酸钠成本仅是乙酸钠的0.2%,淋洗液总成本降低约90%,故选用200 mmol/L NaOH-7.5 mmol/L Na_2_C_2_O_4_进行后续实验。

#### 2.1.2 电位参数的初步选择

表2为目前通用的电位波形,在实际检测中发现,若实验条件不佳或系统被污染,硫化物的色谱峰常拖尾严重,且出现硫化物含量在100 μg/L以下时色谱峰消失的现象。分析原因如下:(1)仪器系统(尤其是色谱柱)被重金属污染;(2)安培检测电位参数不佳(受Ag/AgCl-pH复合参比电极影响),低浓度HS^-^与银电极反应后,会形成Ag_2_S吸附于电极表面影响后续氧化还原反应的发生。因此,施加正相清洗电位(0~0.3 s, -0.1 mV; 0.3~0.4 s, 0.1 mV),信号采集时间为0.2~0.3 s^[18]^,观察低浓度硫化物的出峰稳定性和峰形(见图2)。

**图2:**
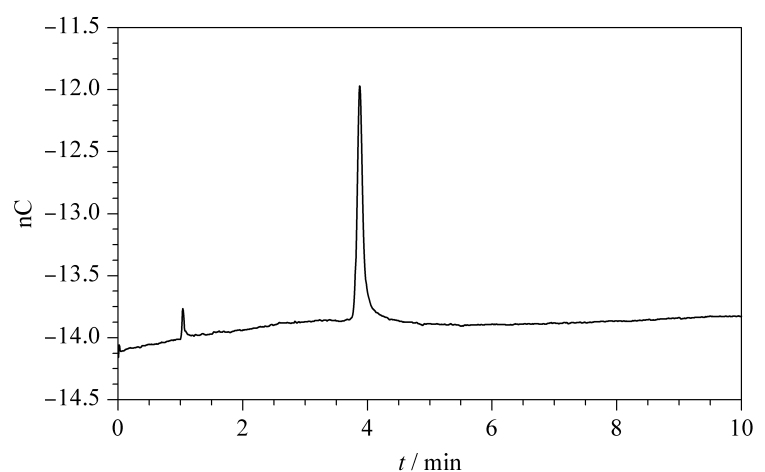
改进电位波形下10 μg/L硫化物的色谱图

由图2所示,使用改进后的电位波形,10 μg/L的硫化物仍可检出,且拖尾问题得到改善。其中峰尾基线的降低是Ag_2_S沉淀后电极表面的电化学恢复,Ag_2_S沉淀被还原,换言之,Ag_2_S沉淀通过产生总体负电流的电势循环而被消除^[18]^。

#### 2.1.3 安培积分时长和波形电位的优化

改进电位下硫化物峰面积较小,不利于降低检出限和应用于实际样品检测,尝试延长积分时间以增加积分定量的准确性,提高灵敏度。设置积分时间0.2、0.3、0.4、0.5、0.6、0.7 s,分析100 μg/L硫化物标准溶液。硫化物在不同积分时长下的峰面积见表3。

**表3:** 硫化物在不同积分时长下的峰面积

Integration time/s	Peak area
0.2	9.4919
0.3	18.8029
0.4	28.3009
0.5	37.4532
0.6	47.9043
0.7	57.3832

由表3可知,积分时间延长,峰面积增大。但积分时间增加,硫化物在银电极上与AgO等银氧化物的反应时间延长,会加剧银电极的消耗^[19,20]^,因此积分时间不宜继续增加,最终选定0.7 s用于积分,0.3 s用于清洗、平衡,以确保电极的稳定,电位波形见表1。

### 2.2 硫化物稳定性研究

在稀释标准溶液的溶剂中分别添加EDTA、EDA和抗氧化剂(抗坏血酸(AsA)、次亚磷酸钠(SHP))以减少硫化物的损失。使用250 mmol/L NaOH、250 mmol/L NaOH-0.2%(质量分数) EDTA、250 mmol/L NaOH-0.5% (质量分数)EDA、250 mmol/L NaOH-5% (体积分数)AsA、250 mmol/L NaOH-5%(体积分数) SHP 5种稳定液将100 mg/L的硫化物标准溶液稀释后避光冷藏储存,于0、1、2、3、4、5、8、12、16 h后检测。结果表明,加入抗坏血酸的硫化物标准溶液出现杂质峰,其他标准溶液中均未出现杂质峰;在EDTA稳定液中,3 h内硫化物含量无明显变化,16 h后仍可保持在90%以上,而其他稳定液中硫化物含量在8 h后降到60%以下。EDTA可络合淋洗液中存在的微量金属离子,减少硫化物损失^[21,22]^。随后选取含EDTA质量分数为0.2%、0.4%、0.5%、0.8%、1.0%的250 mmol/L NaOH溶液将硫化物标准溶液稀释至1000 μg/L,于0、2、4、6、8、10 h后检测,硫化物含量百分比如图3所示。

**图3:**
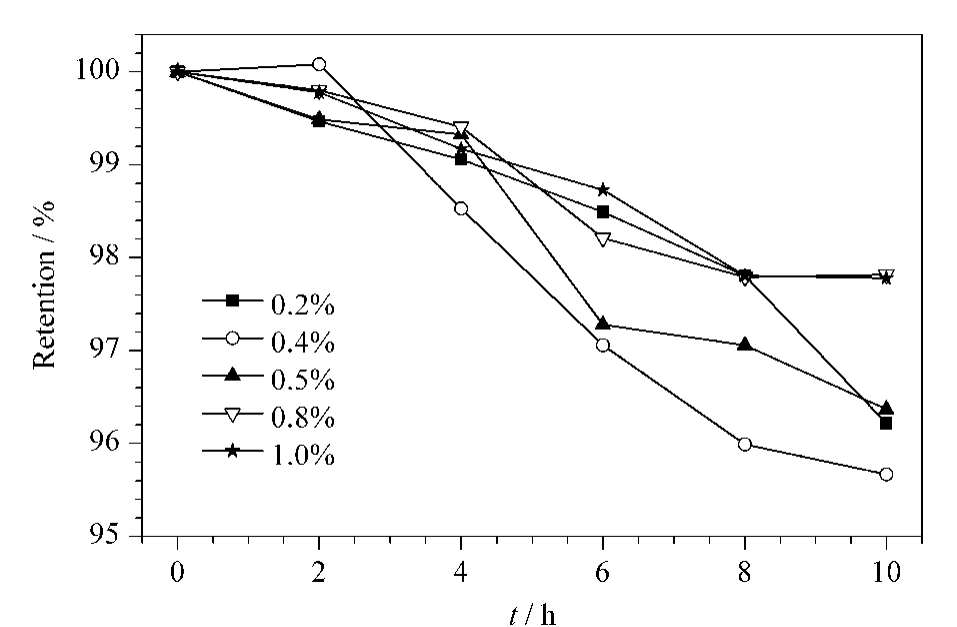
不同质量分数的EDTA对硫化物含量的影响

图3结果表明,10 h内,含EDTA质量分数为0.8%和1.0%的250 mmol/L NaOH溶液中硫化物仅损失2%左右,从效果和使用量考虑,确定使用250 mmol/L NaOH-0.8% EDTA作为硫化物稀释稳定液。

### 2.3 方法学验证

#### 2.3.1 重复性

对配制好的100 μg/L硫化物标准溶液平行进样6次。结果表明,硫化物的峰面积和保留时间的RSD均<0.20%,说明仪器精密度高,重复性好。

#### 2.3.2 线性范围、检出限和定量限

使用250 mmol/L NaOH-0.8% EDTA稳定液将100 mg/L硫化物标准溶液逐级稀释至质量浓度为3000、1000、200、100、50、20、10 μg/L。在1.3节色谱条件下对系列标准溶液依次进样,平行测定3次。以硫化物的质量浓度(*x*, μg/L)为横坐标,色谱峰面积(*y*)为纵坐标进行线性拟合。结果表明,硫化物在10~3000 μg/L范围内线性关系良好,线性方程为*y*=9.789*x*-0.0736,相关系数(*r*^2^)>0.999。以信噪比*S/N*≥ 3确定检出限,以*S/N*≥10确定定量限,分别为1.53 μg/L和5.10 μg/L。与原方法相比,本方法更适用于100 μg/L以下硫化物的分析,检出限和定量限更低,对空气中微量硫化氢气体检测有重要意义。

#### 2.3.3 回收率

使用徽章式被动采样器采集硫化物,对回收率进行验证。向制备好的两组采样器中分别加入200、100 μg/L硫化物标准溶液0.05 mL, 30 min后提取检测,获得被动采样方法回收率(方法回收率=样品中硫化物含量/标准溶液中硫化物含量)。向制备好的采样器中分别加入50000 μg/L硫化物标准溶液0.025、0.05、0.1 mL,得3组加标采样器,与另一组不加标采样器相同条件下采样3天后提取检测,获得实际采样回收率(采样回收率=(加标样品中硫化物含量-未加标样品中硫化物含量)/标准溶液中硫化物含量)。结果表明,在不同水平下方法回收率分别为95.5%和98.9%,采样回收率分别为80.70%、91.23%、80.00%。

### 2.4 实际样品检测

垃圾站是日常生活场所中硫化氢含量较高的区域,由微生物在厌氧条件下分解有机物产生^[23]^。将组装好的被动采样器置于学校两处垃圾处理站,每隔12 h取出一批样品,共采集72 h,测定结果如表4所示。学校垃圾站采样点Ⅱ,被动采样12 h的硫化物的色谱图见图4,可见峰形良好,基线平稳。

**表4:** 学校2处垃圾站点硫化氢的检测结果

Position Ⅰ			Position Ⅱ	
Sampling time/h	Content in the air/(μg/m^3^)		Sampling time/h	Content in the air/(μg/m^3^)
12	9.856		12	5.985
24	6.337		24	4.576
36	8.550		36	6.571
48	7.833		48	7.481
72	7.393		72	5.691

**图4:**
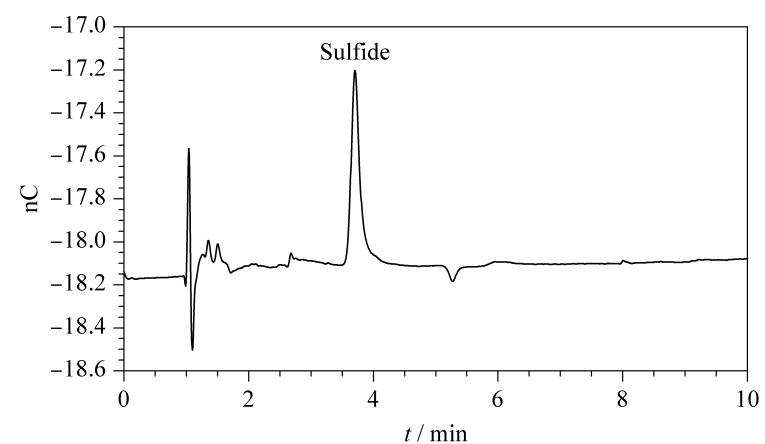
新电位波形下学校垃圾站点Ⅱ采样12 h的硫化物色谱图

从表4可以看出,学校内两个垃圾站点中,采集到硫化氢的含量随时间延长而增加,但均未超过国家规定居民区硫化氢的限值10 μg/m^3[24]^。

## 3 结论

本文针对硫化物常规安培检测中因仪器操作要求高导致实验结果不理想(低于100 μg/L色谱峰易消失、峰拖尾)及硫化物保存稳定性差等问题,提出一整套可靠易操作的解决方法。主要涉及3个方面,首先采用草酸钠替代乙酸钠作为淋洗液的组成,能够大幅降低基线噪声和使用成本,更好掩蔽金属离子以避免干扰;其次,通过修改电位波形,使其更容易检测到低浓度的硫化物,保证峰形对称,避免色谱峰丢失,从而降低实验难度,提高结果可靠性。最后,提出了硫化物保存的新方法,大大延长了硫化物的稳定时间,回收率可达80%以上。综上所述,对原有测试方法改进,使整体实验的成本降低,方法更加简易,效果更好。
